# Association of Cotinine-Verified Cigarette Exposure with Chronic Rhinosinusitis in Korean Adults

**DOI:** 10.3390/ijerph17218291

**Published:** 2020-11-09

**Authors:** Kijeong Lee, In Hak Choi, Hoyoung Lee, Soojeong Choi, Sang Hag Lee, Tae Hoon Kim

**Affiliations:** Department of Otorhinolaryngology-Head & Neck Surgery, College of Medicine, Korea University, Seoul 02841, Korea; peppermint_1111@hotmail.com (K.L.); choi200072@gmail.com (I.H.C.); hyu415@naver.com (H.L.); cjstk4872@naver.com (S.C.); sanghag@kumc.or.kr (S.H.L.)

**Keywords:** chronic rhinosinusitis, cigarette, cotinine level, nasal polyp, total IgE

## Abstract

Chronic rhinosinusitis is known to be influenced by cigarette exposure; however, this relationship is based on the presence of nasal polyps, and objective measurements of cigarette exposure in chronic rhinosinusitis are not well established. This study aimed to estimate the association between chronic rhinosinusitis and smoking status based on self-reported questionnaires and urinary cotinine levels according to the presence of nasal polyps. We analyzed a total of 23,621 participants who participated from the fifth Korea National Health and Nutrition Examination Survey (2010–2012). Serum total and specific IgE level were measured. Higher prevalence of chronic rhinosinusitis with nasal polyps was associated with current smoking status (OR = 1.43, 95% CI = 1.00–2.03). This association was prevalent in participants aged ≤ 50 years (OR = 1.76, 95% CI = 1.01–3.05), and higher urinary cotinine level showed correlation with higher prevalence of chronic rhinosinusitis with nasal polyps in this age group (OR = 1.04, 95% CI = 1.00–1.08). In addition, positive correlation between serum total IgE and urinary cotinine levels was greater in patients with chronic rhinosinusitis (β = 0.493, 95% CI = 0.071–0.916) than in controls (β = 0.062, 95% CI = 0.021–0.103). Aggressive smoking interventions should be performed in patients with chronic rhinosinusitis with nasal polyp, especially in cases of young adults or high serum IgE levels.

## 1. Introduction

Chronic rhinosinusitis (CRS) is an inflammatory condition of the paranasal sinus mucosa characterized by presentation of the following symptoms for at least 12 weeks: nasal obstruction, anterior/posterior discharge, olfactory dysfunction, and facial pain/pressure. The phenotypes associated with CRS are divided into two subsets according to the presence of nasal polyps, and present either as chronic rhinosinusitis with nasal polyps (CRSwNP) and chronic rhinosinusitis without nasal polyps (CRSsNP) [1]. Epidemiologic studies on CRS report its prevalence as 5.2%–27.1%; however, only 25%–30% of these patients have nasal polyps [2,3,4]. Although nasal polyps are classified into two types as eosinophilic and non-eosinophilic, depending on the pathogenesis, CRSwNP has a more severe overall disease burden, often leading to surgery and higher recurrence rates than CRSsNP, regardless of the endotype [5].

Cigarette smoke exposure is one of the most important factors that affects occurrence and the course of CRS, as well as a variety of upper and lower airway tract diseases. Previous pathophysiology studies identified that CRS due to cigarette exposure develops through decreased mucociliary clearance with chloride channel transport loss, goblet cell hyperplasia, tissue remodelling, and changes in biofilms of the nasal cavities [6,7,8,9]. Recently, a systemic review article, reviewing 112 related studies, reported a definite correlation between prevalence of CRS and both active/passive cigarette smoke exposure [10]. In addition, an association study between smoking cessation and CRS reported longer durations of smoking cessation-alleviated sinonasal symptoms in former smokers with CRS [11]. However, it remains unclear which phenotype of CRS is most strongly associated with cigarette exposure. Despite numerous studies related to CRS and cigarettes, few studies have identified its association with objective measurements of nicotine exposure.

The present study aimed to identify an association between cigarette smoke, including both active and passive exposure, with the previously mentioned phenotypes of CRS. In addition to self-reported exposure to cigarettes, we also investigated the association between urinary cotinine levels, an objective measurement of smoking exposure, and CRS, as well as their correlation with atopic conditions associated with CRS.

## 2. Materials and Methods 

### 2.1. Data Collection and Sample Size

This cross-sectional study was performed by collecting data from the fifth National Health and Nutrition Examination Survey (KNHANES V) conducted from January 2010 to December 2012. This nation-wide survey, which was representative of the non-industrialized South Korean population, used a stratified, multistage clustered sampling based on the National Census Data. The survey included a health interview, nutritional survey, and physical examination administered by trained interviewers. The survey sample weights were constructed regarding the survey design, non-response rate, and post-stratification that adjusted for age and sex, in order for the survey population to be representative of the entire South Korean population. In KNHANES V, 150 otorhinolaryngology department residents from 47 institutions participated in the survey and conducted endoscopic ear-nose-throat examinations and medical interviews under standard protocol. All participants provided written informed consent, and the survey protocol was approved by the Institutional Review Board of the Korea Centers for Disease Control and Prevention (2010–02CON-21-C, 2011–02CON-06-C, and 2012–01EXP-01–2C) according to the Ethical Principles for Medical Research Involving Human Subjects, as defined by the Helsinki Declaration. 

**Figure 1:**
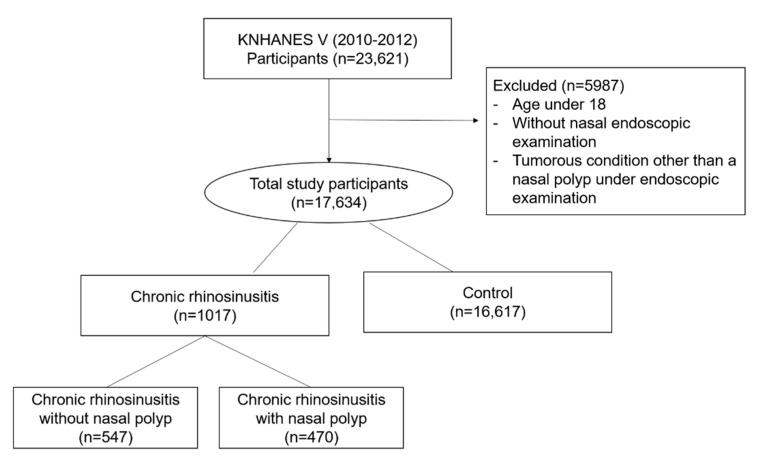
Flow chart of the study population selection. Abbreviation: KNHANES, Korea National and Nutritional Examination Survey; CRS, chronic rhinosinusitis; CRSsNP, chronic rhinosinusitis without nasal polyps; CRSwNP, chronic rhinosinusitis with nasal polyps.

Among the 23,621 participants enrolled in KNHANES V, participants who were under 19 years of age, who did not receive nasal endoscopic examination, and who had tumorous findings in nasal cavity other than polyps were excluded. Thus, a total of 17,634 participants were analyzed in the study (Figure 1). All of participants included in the study were those who answered questionnaires about active and passive smoking status, however, in more detailed questions about passive smoking, only 10,335 and 2267 responded to the questions of exposure in the office and at home, respectively. Furthermore, in order to evaluate association with CRS and an objective indicator of smoking exposure, additional analysis was performed on 1916 participants who underwent urinary cotinine level examination. 

### 2.2. Assessment of Medical Conditions

Questionnaires related to the CRS symptoms included four symptoms, anterior/posterior nasal drip, nasal obstruction, facial pain/pressure, and olfactory dysfunction for at least three months. CRS was diagnosed when more than two of four symptoms related to CRS with at least one of either anterior/posterior nasal drip or nasal obstruction were present or nasal polyp was identified by nasal endoscopic examination. CRSsNP and CRSwNP were classified according to the endoscopic findings of nasal polyps. 

Comorbidities including asthma and allergic rhinitis were defined based on the self-reported experience of being diagnosed by a physician, and nasal septal deviation was defined according to the endoscopic examination. 

Since 10% of the participants who participated in KNHANES in 2010 underwent ImmunoCAP100 (Thermo Scientific; Uppsalsa, Sweden), data on serum total IgEs and serum specific IgEs for three common indoor allergens (Derma~farina, cockroaches, and dogs) were also collected from these subjects. The cutoff value of serum total IgEs and specific IgEs were defined as 100 kU/L and 0.35 kU/L, respectively. 

### 2.3. Assessment of Cigarette Exposure and Other Socioeconomic Variables 

Questionnaires related to active smoking included smoking status-relevant questions such as “Do you currently smoke?” and “How many cigarettes have you smoked in total?” to gain a detailed understanding of their smoking habits. Participants who answered that they have smoked less than 100 cigarettes in their lifetime were classified as never smokers. Participants who answered that they do not currently smoke but have smoked more than 100 cigarettes were categorized as ex-smokers. Participants who answered that they currently smoke and had smoked more than 100 cigarettes were defined as current smokers. Furthermore, participants were asked about their secondhand smoke exposure at work or home, and the duration of exposure. In addition, urinary cotinine levels (ng/mL) were measured in 10% of total participants who enrolled KNHANES 2010–2012. 

Socioeconomic variables regarding age, sex, residency, household income, education, alcohol consumption, and cigarette exposure were collected and analyzed. Participants were classified into an urban group and a rural group based on residency, into four groups by quartile based on the household income, into four groups based on the education level as less than elementary school, less than high school, less than college, and more than college. Participants were categorized into two groups by frequency of alcohol consumption as follows: twice a week or less, or more than twice a week during the past year. 

### 2.4. Statistical Analysis 

Statistical Package for the Social Science (SPSS) version 20 (IBM Corp., Armonk, NY, USA) complex sample module was used to perform statistical analyses. To estimate the general South Korean populations from the data, KNHANES sampling weight variables were used. To compare the characteristics of participants according to groups, continuous variables were described as the mean and standard error (SE), and the statistical differences between groups were analyzed by a t-test under the complex sample module. As for categorical variables, the description was done using the number and percentage, and the difference was analyzed by the chi-square analysis. Logistic regression analysis was performed to analyze the association between cigarette exposure and CRS, and the odds ratio (OR) and 95% CIs were calculated. For multivariable analysis, confounding factors for adjustment included age, sex, household income, educational level, residency, alcohol consumption, allergic rhinitis and nasal septal deviation. Pearson’s correlation coefficient was used to analyze the correlation between urinary cotinine level and smoking-related factors (average numbers of cigarettes, passive smoking exposure duration at office, passive smoking exposure duration at home) as well as serum IgE level.

## 3. Results 

### 3.1. General Characteristics of Participants

Of the total 17,634 participants (mean age: 45.60 ± 0.24), 1017 (5.94 ± 0.28%) were categorized in the chronic rhinosinusitis group (mean age: 46.38 ± 0.72) (Table S1). According to the presence of nasal polyp, 547 participants were classified to have CRSsNP, and 470 participants (3.5 ± 0.23%) were classified to have CRSwNP (2.5 ± 0.16%) (Table 1). 

For factors related to cigarette exposure, higher rate of smoking experience was identified in the CRS group (current smoker: 29.2%, ex-smoker: 20.5%) compared to the controls (current smoker 25.7%, ex-smoker 17.0%), and participants with CRSwNP showed higher proportions of positive smoking status (current smoker: 32.1%, ex-smoker: 23.3%) (Table 1). 

In addition, urinary cotinine levels were higher in the CRS group (525.45 ± 115.79) compared to the control group (488.16 ± 26.41). However, when analyzed according to the presence of polyps, the CRSwNP group (755.71 ± 215.09) clearly showed higher urinary cotinine levels compared to the controls, whereas the CRSsNP group showed lower urinary cotinine level (380.56 ± 115.55).

### 3.2. Association between Self-Reported Smoking Status and Chronic Rhinosinusitis

In univariable logistic regression analysis for the association between CRS and active smoking, CRS was identified to be associated with smoking status, showing higher prevalence in current smokers (OR = 1.29, 95% CI = 1.07–1.56) as well as in the ex-smoker group (OR = 1.37, 95% CI = 1.10–1.72) (Table 2). The result of further analysis, according to the presence of nasal polyps, showed higher prevalence of both CRSsNP (OR = 1.60, 95% CI = 1.21–2.13) and CRSwNP (OR = 1.76, 95% CI = 1.31–2.37) in the ex-smoker group. After adjustment for confounding factors, the prevalence of CRSwNP was shown to be higher in current smokers (OR = 1.43, 95% CI = 1.00–2.03). 

As for passive smoking, although the overall presence of secondhand smoking either at the office or at home showed no meaningful correlation with prevalence of CRS, participants exposed to passive smoke at the office for over 1 h a day were shown to have higher chances of CRS in both univariable (OR = 1.36, 95% CI = 1.00–1.86) and multivariable (OR = 1.42, 95% CI = 1.04–1.95) analysis (Table 2). This correlation was not identified in further analysis according to the CRS phenotype classified based on the presence of nasal polyps. 

To evaluate the association between objective measurement of nicotine exposure and prevalence of CRS, urinary cotinine level for 1824 participants were collected. Logistic regression analysis for overall participants showed no definite correlation between urinary cotinine and each phenotype of CRS (Table 2).

### 3.3. Association between Factors Related to Cigarette Exposure and Chronic Sinusitis According to Age

In a subgroup analysis according to the age, only current smokers of participants aged 50 or below were associated with higher prevalence of CRSwNP after adjustment of confounding factors (OR = 1.76, 95% CI = 1.01–3.05) (Table 3). As for passive smoking, a correlation between passive smoking at the office for more than 1 h a day and higher prevalence of CRS was identified in participants aged more than 50 years of age (OR = 1.70, 95% CI = 1.06–2.72). 

Higher urinary cotinine level was associated with prevalence of CRSwNP in participants aged 50 or younger (OR = 1.04, 95% CI = 1.00–1.08) (Table 3). Furthermore, Pearson’s correlation coefficient was conducted to identify the correlation between urinary cotinine level and amount of active/passive cigarette exposure (Table S2). For current smokers, the average number of cigarettes smoked showed stronger correlation with urinary cotinine level in participants aged 50 years old or younger (r = 0.453, 95% CI = 0.422–0.648), even though correlation between these factors was identified in participants older than 50 as well (r = 0.303, 95% CI = 0.175, 0.558). As for passive smoking, hours for exposure to other’s cigarette smoking at the office nor home showed meaningful correlation with urinary cotinine level.

### 3.4. Association between Urinary Cotinine Level and Serum Immunogolbulin E

Since atopic conditions are known to affect the symptom severity and disease prognosis in CRS patients, we investigated whether urinary cotinine level is correlated with serum total IgE and specific IgEs for three aeroallergens. 

**Figure 2:**
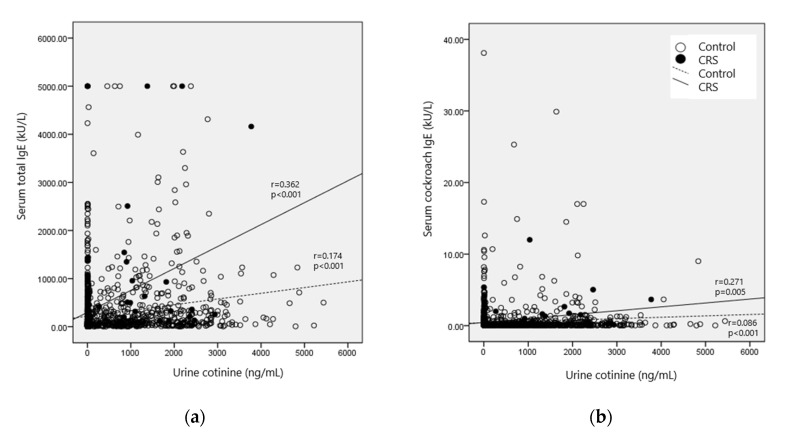
Pearson’s correlation coefficient between the urinary cotinine level and (**a**) serum total Immunoglobulin E (IgE); (**b**) serum cockroach IgE levels.

Higher urinary cotinine level showed correlation with elevated serum total IgE level in healthy participants (r = 0.174, 95% CI = 0.123–0.213); however, this correlation was more prominent in the CRS group (r = 0.362, 95% CI = 0.294–0.997). As for serum specific IgEs for three aeroallergens, only cockroach allergen showed a positive correlation with the urinary cotinine level, and this correlation was shown to be clinically meaningful only in the CRS group (r = 0.271, 95% CI = 0.074–0.510) (Figure 2, Table S3).

Further dose–response relationships between serum total IgE, serum cockroach IgE, and urinary cotinine levels using multiple linear regression after adjustment for confounding factors were analyzed. Serum total IgE levels increased with increasing urinary cotinine levels in both the control (β = 0.062, 95% CI = 0.021, 0.103) and the CRS group (β = 0.493, 95% CI = 0.071–0.916), with a greater correlation identified in the CRS group.

## 4. Discussion

In this cross-sectional study using nation-wide survey data, active smoking status was closely related with CRSwNP in younger adults aged below 50, while no meaningful association was identified in either the CRSwNP in older adults or in CRSsNP. This finding was consistent with the association between urinary cotinine and CRS. In addition, the serum urinary cotinine level was correlated with higher serum total IgE levels, and this correlation was stronger in CRS participants compared to the control. 

Numerous studies have investigated the effect of cigarette smoke exposure on CRS and most of them are based on a self-reported questionnaire. In this nationwide survey-based study, we used urinary cotinine levels and self-reported smoking status to identify the association between smoking and CRS. Cotinine, as an objective indicator of cigarette smoke exposure, has been rarely used in assessments, although it is a sound objective determinant of smoking. A major metabolite of nicotine, cotinine is considered a reliable biomarker reflecting smoking conditions because of its longer half-life (16–20 h) than nicotine (2 h) [12,13]. Cotinine can be detected in body fluids including saliva, blood, and urine, as well as hair and nails, with a higher concentration compared to nicotine. In this study, urinary cotinine levels showed correlation with the average number of cigarettes per day in current smokers; however, its measurements in secondhand smoking were poorly correlated.

A previous study on the association between cigarette exposure and CRS using the NHANES in the United States reported no definite correlation between serum cotinine concentration and CRS, despite findings that current smoking status was related to increased risk of chronic sinusitis [14]. In this study, increased prevalence of CRSwNP was associated with current smoking status, especially in participants under the age of 50, and its correlation with urinary cotinine level showed consistent results. A recent investigation on nasal polyps via E-prostanoid (EP) receptors identified that cigarette exposure stimulates the production of prostaglandin E2 and proinflammatory cytokines in CRSwNP patients through EP2 and EP4 downregulation [15]. Furthermore, an experimental study examined the impact of smoking on sinonasal dendritic cell (sDCs) subsets in CRSwNP, reporting that cigarette smoke extracts stimulate inflammatory cytokine production from human sinonasal epithelial cells and induces DC maturation, migration, and co-stimulatory molecule expression [16]. Since CRSwNP is a Th2-associated disease and DCs are important for the Th2 response, the effect of smoking is suspected to be prominent in CRSwNP by acting on DCs. The previous studies reporting that cigarettes induce Th2-skewed airway inflammations and that younger CRSwNP patients have type 2 inflammatory endotype more often compared to the elderly support our results of a greater association between smoking and CRSwNP under 50 years of age [17,18]. On the other hand, the prevalence of CRSsNP showed no clinical association with active smoking status. Although additional experimental studies should be conducted, this might be related to the components of active smoke that acts on edema or inflammatory mediators leading to cellular transformations and causes cellular division forming nasal polyps, compared to fibrotic or collagen deposition within the nasal mucosa in CRSsNP pathogenesis [5]. 

As for secondhand smoking, literature on the correlation with CRS shows conflicting results. Tammemagi et al. reported that overall experience of passive smoking was associated with increased prevalence of CRS; however, this association was relevant only in workplaces and private places, but not in household exposure [19]. Furthermore, a result from the NHANES with 20,050 participants aged over 17 showed no definite association between secondhand smoking and sinusitis; however, this investigation only considered passive smoke exposure at home [14]. Our study also suggested only secondhand smoke exposure over an hour at the office was associated with higher prevalence of CRS, whereas household exposure was not. However, this result might be due to the relatively small number of subjects analyzed for secondhand smoke exposure, including 2267 participants, especially 139 for the CRS group. 

Increased levels of serum total IgE according to active and passive cigarette exposure have been reported by several population-based studies; however, this correlation in CRS patients is not yet studied despite the clinical importance of serum total IgE levels in its association with CRS symptoms severity, treatment response, and disease prognosis [20,21]. The analysis from the present study showed an association between urinary cotinine level and serum total IgE levels, even after adjustment for confounding factors, and this association was much stronger in CRS patients. This result shows consistency with the previous study between smoking and total IgE in asthma patients, reporting total IgE levels in current smokers as 3.5 times greater than the “never smoked with asthma” group, whereas only a 1.5 times difference was identified in the control group, respectively [22]. Chronic exposure to cigarettes shows neutrophilic inflammation mediated by T17 inflammation in lower respiratory tract disease [23,24]. In cases of CRS, two recent experimental studies of Huang et al. reported increased expression of Interleukin(IL)-17A in nasal tissues of smokers and its correlation with poorer improvement for asthmatic patients accompanying CRS after endoscopic sinus surgery in smoking populations [25,26]. Moreover, the role of IL-17A in production of IgE and exaggeration of this activity of IL-17A in allergic patients supports our results that the correlation between urinary cotinine and total IgE level was greater in CRS group, which has a larger proportion of participants with allergic rhinitis, compared to the control [27]. This might also support the more pronounced correlation between current smoking and CRSwNP in the younger adult group, as our data showed higher prevalence of allergic rhinitis in the CRSwNP group under 50 years of age (24.9 ± 4.3%) than in those over 50 years of age (10.7 ± 2.2%) (*p* = 0.001). 

Our study had several limitations; firstly, a causal relation between CRS and smoking was difficult to determine because of the cross-sectional design of the data. In addition, the effects of secondhand smoking exposure on CRS for each active smoking status could not be investigated because the statistical analysis was difficult to conduct due to missing data when the groups were subdivided. Urinary cotinine levels were also measured only in a small proportion of the total participants, and the possible effect of other tobacco products or electronic cigarettes could not be determined since KNHANES V did not included related questionnaires. Nevertheless, the strength of this study is that the correlation between CRS and cigarette exposure was determined through objective measurements based on nasal endoscopic examination, urinary cotinine level, and serum IgE levels, using nation-wide survey data.

## 5. Conclusions

In conclusion, current smoking status was associated with higher prevalence of CRSwNP and the correlation predominantly within young adults. Additionally, correlation with higher serum IgE according to increased urinary cotinine level might be associated with the development of the disease. The findings from this nation-wide study emphasize that among two phenotypes of CRS, more aggressive interventions should be made with cigarettes in patients with nasal polyps, especially when patients are young adults or when the patients show high serum IgE level in a clinical setting. 

## Figures and Tables

**Table 1:** Status of cigarette exposure-related factors in the control and the chronic rhinosinusitis groups.

Variables	Control (n = 16,617, N = 97,408,503)	CRS (n = 1017, N = 6,463,837)	CRSsNP (n = 547, N = 3,917,093)	CRSwNP (n = 470, N = 2,746,744)
Active smoking status				
Never smoker (%)	10,267 (57.2)	552 (50.3)	327 (54.4)	225 (44.6)
Current smoker (%)	3349 (25.7)	239 (29.2)	114 (27.1)	125 (32.1)
Ex-smoker (%)	3001 (17.0)	226 (20.5)	109 (18.6)	117 (23.3)
Passive smoking status				
None (%)	10,613 (57.4)	631 (54.6)	328 (52.1)	303 (58.1)
Yes (%)	6004 (42.6)	386 (45.4)	219 (47.9)	167 (41.9)
Office < 1 h ^1^	3351 (38.4)	199 (36.1)	107 (43.5)	92 (35.1)
Office ≥ 1 h ^1^	1020 (12.1)	86 (16.0)	50 (21.8)	36 (15.4)
Home < 1 h ^2^	1411 (62.8)	76 (50.1)	50 (48.6)	26 (53.5)
Home ≥ 1 h ^2^	326 (15.1)	24 (22.9)	17 (24.5)	7 (19.1)
Urinary cotinine, ng/mL (SE) ^3^	488.16 ± 26.41	525.45 ± 115.79	380.56 ± 115.55	755.71 ± 215.09

Abbreviation: KNHANES, Korea National Health and Nutrition Examination Survey; CRS, chronic rhinosinusitis; CRSsNP, chronic rhinosinusitis without nasal polyps; CRSwNP, chronic rhinosinusitis with nasal polyps, n = unweighted number of study population, N = weighted number of study population.^1^ 10,335 of the total participants and 969 of the CRS participants answered the questions, ^2^ 2267 of the total participants and 139 of the CRS participants answered the questions, ^3^ 1824 of the total participants and 92 of the CRS participants underwent the examination.

**Table 2:** Association between the self-response cigarette exposure status and chronic sinusitis in KNHANES V (2010–2012).

Variables	CRS		CRSsNP		CRSwNP	
	Crude OR (95% CI)	Adjusted OR ^1^ (95% CI)	Crude OR (95% CI)	Adjusted OR ^1^ (95% CI)	Crude OR (95% CI)	Adjusted OR ^1^ (95% CI)
Active smoking status						
Never smoker (%)	1 (ref)	1 (ref)	1 (ref)	1 (ref)	1 (ref)	1(ref)
Current smoker (%)	1.29 (1.07,1.56)	1.22 (0.95,1.58)	1.11 (0.86,1.43)	1.11 (0.79,1.55)	1.15 (0.85,1.56)	1.43(1.00,2.03)
Ex-smoker (%)	1.372 (1.10,1.72)	1.11 (0.84,1.48)	1.60 (1.21,2.13)	1.16 (0.80,1.70)	1.76 (1.31,2.37)	1.18 (0.83,1.67)
Passive smoking status						
None (%)	1 (ref)	1 (ref)	1 (ref)	1 (ref)	1 (ref)	1 (ref)
Yes (%)	1.12 (0.94,1.34)	1.13 (0.94,1.36)	1.24 (0.97,1.57)	1.15 (0.89,1.47)	0.97 (0.76,1.24)	1.13 (0.87,1.48)
Office < 1 h	0.97 (0.77,1.21)	0.96 (0.76,1.23)	1.01 (0.75,1.37)	0.94 (0.68,1.28)	0.91 (0.67,1.25)	1.02 (0.73,1.44)
Office ≥ 1 h	1.36 (1.00,1.86)	1.42 (1.04,1.95)	1.43 (0.98,2.10)	1.42 (0.95,2.11)	1.28 (0.79,2.07)	1.46 (0.90,2.36)
Home < 1 h	0.63 (0.40,1.01)	0.62 (0.37,1.03)	0.62 (0.35,1.10)	0.62 (0.33,1.16)	0.67 (0.31,1.44)	0.67 (0.29,1.58)
Home ≥ 1 h	1.26 (0.66,2.41)	1.16 (0.59,2.30)	1.35 (0.64,2.89)	1.33 (0.60,2.97)	1.03 (0.33,3.19)	0.94 (0.28,3.15)
Urinary cotinine, ng/mL (SE)	1.00 (0.78,1.03)	1.00 (0.97,1.04)	0.98 (0.95,1.03)	0.98 (0.94,1.03)	1.03 (0.99,1.06)	1.02 (0.99,1.06)

Abbreviation: KNHANES, Korea National Health and Nutrition Examination Survey; CRS, chronic rhinosinusitis; CRSsNP, chronic rhinosinusitis without nasal polyps; CRSwNP, chronic rhinosinusitis with nasal polyps; OR, odds ratio; CI, confidence interval. ^1^ Adjusted for age, sex, residency, household income, education, alcohol consumption, asthma, allergic rhinitis, and nasal septal deviation.

**Table 3:** Adjusted odds ratio for the association between cigarette exposure-related factors and chronic rhinosinusitis according to the age subgroup

	Adjusted OR ^1^ (95% CI)					
	Age ≤ 50			Age > 50		
Variables	CRS	CRSsNP	CRSwNP	CRS	CRSsNP	CRSwNP
Active smoking status						
Never smoker (%)	1 (ref)	1 (ref)	1 (ref)	1 (ref)	1 (ref)	1 (ref)
Current smoker (%)	0.79 (0.57,1.09	1.09 (0.75,1.57)	1.76 (1.01,3.05)	0.87 (0.59,1.28)	1.31 (0.71,2.44)	1.05 (0.66,1.66)
Ex-smoker (%)	0.87 (0.58,1.30)	1.15 (0.72,1.85)	1.16 (0.62,2.20)	0.96 (0.66,1.41)	1.09 (0.60,1.98)	1.00 (0.63,1.58)
Passive smoking status						
None (%)	1 (ref)	1 (ref)	1 (ref)	1 (ref)	1 (ref)	1 (ref)
Yes (%)	0.93 (0.72,1.20)	1.15 (0.85,1.57)	0.91 (0.59,1.40)	0.80 (0.62,1.03)	1.23 (0.81,1.86)	1.28 (0.94,1.75)
Office < 1 h	0.91 (0.66,1.26)	0.94 (0.64,1.38)	0.86 (0.51,1.44)	1.12 (0.80,1.56)	0.94 (0.53,1.66)	1.24 (0.83,1.85)
Office ≥ 1 h	1.32 (0.86,2.02)	1.25 (0.77,2.04)	1.44 (0.70,2.97)	1.70 (1.06,2.72)	2.07 (0.98,6.39)	1.45 (0.82,2.56)
Home < 1 h	0.54 (0.28,1.01)	0.58 (0.29,1.18)	0.41 (0.08,2.05)	0.91 (0.43,1.90)	0.97 (0.28,3.37)	0.88 (0.36,2.12)
Home ≥ 1 h	1.13 (0.49,2.61)	1.28 (0.51,3.21)	0.77 (0.09,6.63)	1.36 (0.45,4.07)	1.82 (0.37,8.98)	1.09 (0.28,4.22)
Urinary cotinine, ng/mL (SE)	0.98 (0.95,1.02)	0.99 (0.94,1.05)	1.04 (1.00,1.08)	1.03 (0.98,1.10)	0.96 (0.89,1.05)	0.97 (0.90,1.04)

Abbreviation: CRS, chronic rhinosinusitis; CRSsNP, chronic rhinosinusitis without nasal polyps; CRSwNP, chronic rhinosinusitis with nasal polyps; OR, odds ratio; CI, confidence interval. ^1^ Adjusted for age, sex, residency, household income, education, alcohol consumption, asthma, allergic rhinitis, and nasal septal deviation.

## Supplementary Materials

The following are available online at https://www.mdpi.com/1660-4601/17/21/8291/s1, Table S1: Baseline characteristics of controls and participants with chronic laryngitis in KNHANES V (2010–2012). Table S2: Pearson’s correlation analysis between cigarette exposure and urinary cotinine levels according to age subgroups Table S3: Pearson’s correlation analysis between urine cotinine and serum total/specific IgE levels
