# Supplementary material for: Association of Cotinine-Verified Cigarette Exposure with Chronic Rhinosinusitis in Korean Adults
Source: Int J Environ Res Public Health. 2020 Nov 9;17(21):8291. doi: 10.3390/ijerph17218291 (PMC7665152; doi:10.3390/ijerph17218291)
Supplement: Supplementary file 1 [file ijerph-17-08291-s001.pdf]

International Journal of
Environmental Research and Public Health

Table S1. Baseline characteristics of controls and participants with chronic laryngitis in KNHANES V (2010–2012)

	Unweighted number (Weighted %)		
**Variables**	**Control (n = 16,617; N = 97,408,503)**	**CRS (n = 1,017; N = 6,463,837)**	**P-value**
Age (SE)	45.6 ± 0.243	46.38 ± 0.715	0.275
Sex, male	7026 (49.1)	503 (56.4)	0.001
Residency, urban	13174 (80.3)	761 (74.0)	0.002
Household income			0.373
<25%	3251 (16.2)	232 (18.4)	
25–50%	4208 (27.1)	269 (28.5)	
50–75%	4447 (29.1)	264 (26.8)	
>75%	4495 (27.6)	244 (26.3)	
Education level			0.124
Less than elementary	4157 (18.8)	286 (21.1)	
Less than high school	1766 (10.2)	126 (11.1)	
Less than college	5392 (38.8)	321 (39.9)	
More than college	4855 (32.2)	253 (28.0)	
Alcohol consumption more than twice/week (%)	8430 (58.5)	513 (58.1)	0.851
Asthma	702 (3.9)	94 (8.8)	<0.001
Allergic rhinitis (%)	1987 (13.2)	337 (37.0)	<0.001
Nasal septal deviation (%)	8042 (50.5)	486 (52.5)	0.341

n, unweighted number of study population; N, weighted number of study population; CI, confidence interval; KNHANES V, Korea National Health and Nutrition Examination Survey, Fifth; SE< standard error

[1] 1,824 of the total participants and 92 of the CRS participants underwent the examination

Table S2. Pearson's correlation analysis between cigarette exposure and urinary cotinine levels according to age subgroups

Cigarette exposure/day	Age ≤ 50			Age > 50		
	**n**	**r**	**95% CI**	**n**	**r**	**95% CI**
Average number of cigarettes[1]	339	0.453	(0.422, 0.648)	143	0.303	(0.175, 0.558)
Passive smoking at office (h)	110	0.105	(−0.097, 0.344)	46	−0.100	(−0.305, 0.140)
Passive smoking at home (h)	26	−0.198	(−0.675, 0.238)	16	−0.223	(−0.694, 0.338)

n, unweighted number of study population; r, Pearson's correlation coefficient; CI, confidence interval

[1] Current smoker

Table S3. Pearson's correlation analysis between urine cotinine and serum total/specific IgE levels

	Control			CRS		
	**n**	**r**	**95% CI**	**n**	**r**	**95% CI**
Serum total IgE	1721	0.174	(0.123–0.213)	92	0.362	(0.294–0.997)
Serum specific IgE						
Dermatophagoides farina	1719	0.043	(−0.004–0.087	92	0.147	(−0.066–0.368)
Cockroaches	1718	0.086	(0.038–0.127)	91	0.271	(0.074–0.510)
Dogs	1719	0.002	(−0.046-0.050)	91	0.108	(−0.110–0.342)

CRS, chronic rhinosinusitis; n, unweighted number; r, Pearson's correlation coefficient

CI, confidence interval, IgE, immunoglobulin E
